# Exploring the Origin of Maximum Entropy States Relevant to Resonant Modes in Modern Chladni Plates

**DOI:** 10.3390/e24020215

**Published:** 2022-01-29

**Authors:** Yu-Hsin Shu, Yu-Chen Tseng, Yu-Hsiang Lai, Yan-Ting Yu, Kai-Feng Huang, Yung-Fu Chen

**Affiliations:** Department of Electrophysics, National Yang Ming Chiao Tung University, Hsinchu 30010, Taiwan; yuhsin.sc10@nycu.edu.tw (Y.-H.S.); taylor814cebycheng.sc08@nycu.edu.tw (Y.-C.T.); nctu0652051.sc06@nycu.edu.tw (Y.-H.L.); yentingyu0204@nycu.edu.tw (Y.-T.Y.); kfhuang@cc.nctu.edu.tw (K.-F.H.)

**Keywords:** maximum entropy, pattern formation, point interaction, modern Chladni plates

## Abstract

The resonant modes generated from the modern Chladni experiment are systematically confirmed to intimately correspond to the maximum entropy states obtained from the inhomogeneous Helmholtz equation for the square and equilateral triangle plates. To investigate the origin of maximum entropy states, the inhomogeneous Helmholtz equation is modified to consider the point interaction coming from the driving oscillator. The coupling strength associated with the point interaction is characterized by a dimensionless factor *α*. The *δ* potential of the point interaction is numerically modelled by a truncated basis with an upper index *N*. The asymptotic behavior for the upper index *N* is thoroughly explored to verify that the coupling strength of *α* = 1.0 can make the theoretical resonant modes agree excellently with the maximum entropy states as N→∞. It is further authenticated that nearly the same resonant modes can be obtained by using a larger coupling strength *α* when a smaller upper index *N* is exploited in the calculation.

## 1. Introduction

Ernst Chladni in the 18th century demonstrated a classical experiment related to acoustic phenomenon by means of vibrating a thin plate with a bow and manifesting the resonant nodal-line pattern with small sands [1,2]. Chladni figures have stimulated numerous scientific explorations in physics including quantum chaos [3], self-organization of granular media [4,5], microscale acoustofluidics [6,7], and pattern formation [8,9]. The classical Chladni figures obtained by a bow are found to considerably different from the resonant nodal-line patterns in the modern Chladni plate which is driven by a mechanical oscillator whose vibrating frequency can be precisely controlled to generate the resonant modes. Thanks to the advantages of high precision, reproducibility, and simple performing, the modern Chladni plate is a promising experiment for creating advanced applications such as automated patterning of micro-objects [10,11], non-contaminated positioning of biomolecules [12,13], and sorting different particles [14,15].

The formation of modern Chladni patterns has been successfully elucidated with the inhomogeneous Helmholtz equation and the maximum entropy principle [16,17,18,19,20]. The coincidence of the experimental resonant frequencies and the maximum entropy states can be comprehended from the principle of energy equipartition in statistical mechanics. Nevertheless, it is of great importance to explore the origin of the resonant modes in modern Chladni plates for progressive developments.

In this work, we systematically identify the intimate correspondence between the resonant modes generated from the modern Chladni experiment and the maximum entropy states solved from the inhomogeneous Helmholtz equation for the square as well as equilateral triangle plates. The point interaction arising from the driving oscillator is considered in the inhomogeneous Helmholtz equation to explore the origin of maximum entropy states. A dimensionless factor *α* is used to characterize the coupling strength associated with the point interaction. Moreover, a truncated basis with an upper index *N* is employed to model the *δ* potential of the point interaction. We numerically verify that as N→∞, the coupling strength of *α* = 1.0 can make the theoretical resonant modes agree excellently with the maximum entropy states. We further confirm that when a smaller upper index *N* is used in the calculation, a larger *α* can be effectively exploited to obtain almost the same theoretical resonant modes. The developed model with the point interaction is believed to be useful for creating novel applications. The whole structure of this paper includes the experimental results for resonant frequencies and modes of modern Chladni plates, the theorical maximum entropy states, and the point-interaction model for exploring the origin of maximum entropy states relevant to modern Chladni plates.

## 2. Frequency Spectrum and Resonant Modes

**Figure 1:**
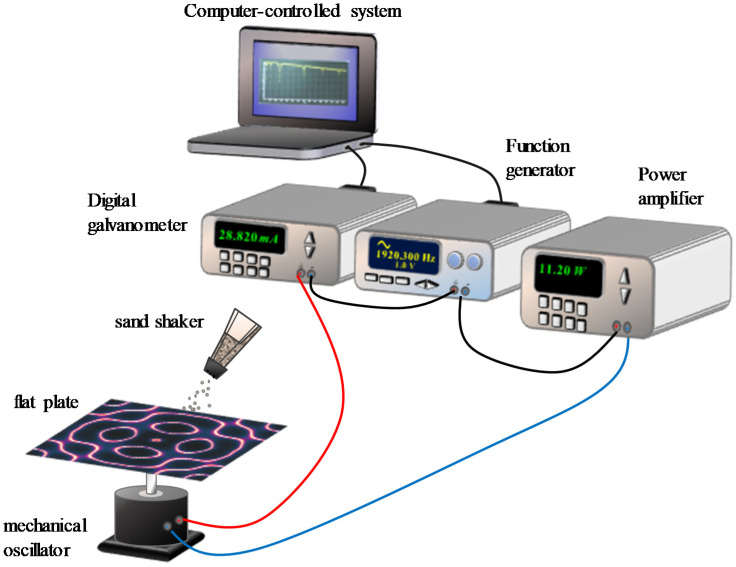
Experimental scheme for the modern Chladni plate driven with an electronically controlled mechanical oscillator.

Figure 1 depicts the experimental scheme for the modern Chladni plate driven with an electronically controlled mechanical oscillator [16]. A sinusoidal function generator with an automatic scanning system was designed to measure the spectrum of resonant frequencies with a resolution of 0.1 Hz. Two different plates were used to investigate the resonant Chladni figures. One is a square plate with a side length of 320 mm; the other is an equilateral triangle plate with a side length of 365 mm. Two thin plates were manufactured with aluminum sheets with a thickness of 1 mm. The center of the thin plate was fixed at the mechanical oscillator with a screw supporter. The mechanical oscillator was excited with an amplified sinusoidal voltage source for the frequency ranging from 200 to 5000 Hz. A digital galvanometer was connected in series to the mechanical oscillator to measure the electric current through the vibrating system. Since the resonance causes the electric resistance of the vibrating system to increase abruptly, the resonant frequencies can be precisely determined via probing the change in the electric resistance of the system [16]. We employed silica sand of 0.3 mm grain size to exhibit Chladni figures at resonant frequencies and exploited a digital camera to store the experimental images.

**Figure 2:**
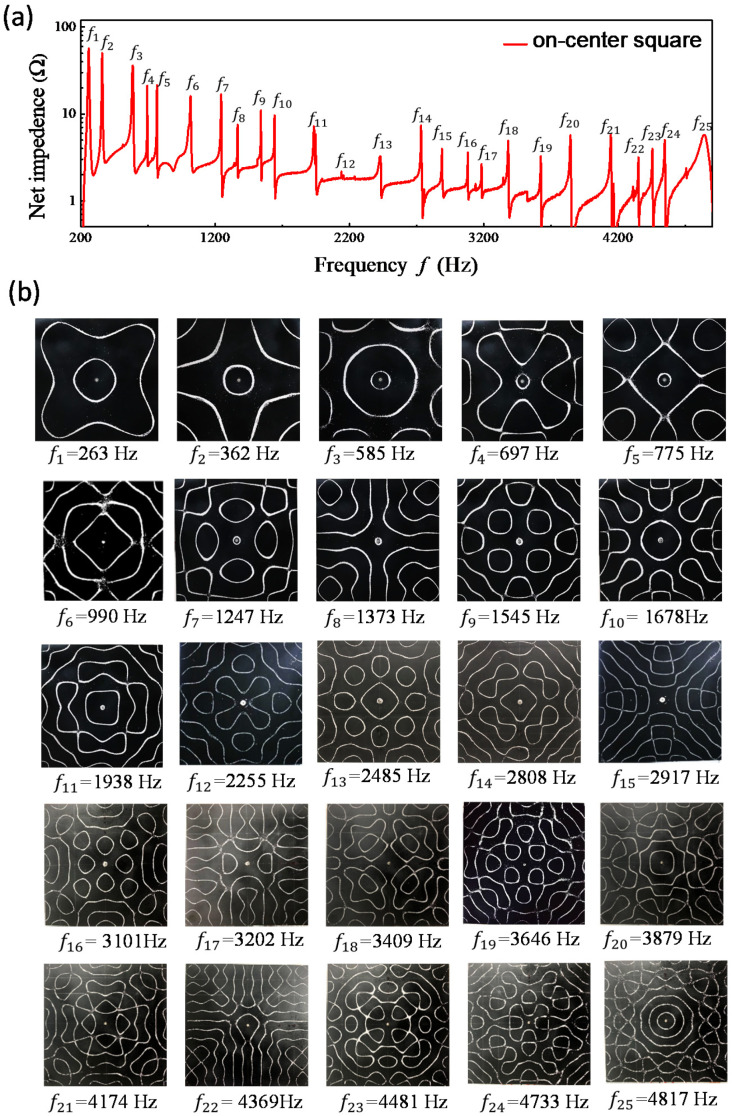
(**a**) Experimentally measured spectrum for the variation of the resistances as a function of the driving frequency in the vibrating square plate. (**b**) Experimental nodal-line patterns of the resonant modes.

**Figure 3:**
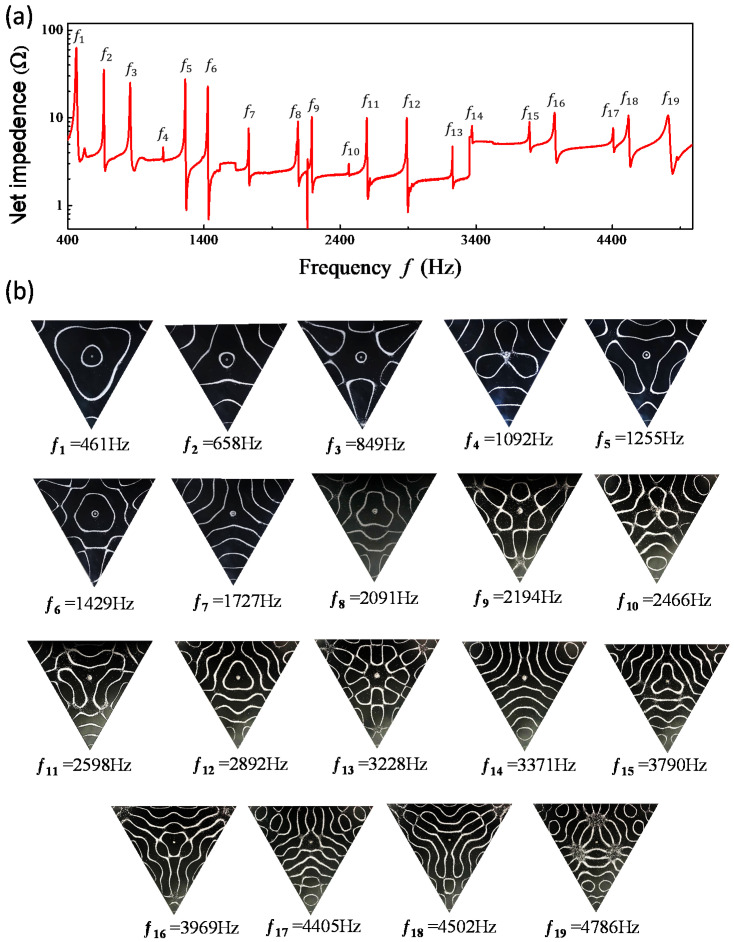
(**a**) Experimentally measured spectrum for the variation of the resistances as a function of the driving frequency in the vibrating equilateral triangle plate. (**b**) Experimental nodal-line patterns of the resonant modes.

Figure 2a shows the measured spectrum for the variation of the resistances as a function of the driving frequency in the vibrating square plate. The resonant frequencies can be precisely identified from the peaks of the frequency spectrum. The nodal-line patterns of the resonant modes are shown completely in Figure 2b. All nodal-line patterns can be found to be obviously distinct from the chessboard structures of eigenmodes. Experimental results for the frequency spectrum and individual resonant nodal-line patterns obtained with the equilateral triangle plate are shown completely in Figure 3a,b. Like the results for the square plates, resonant nodal-line patterns are dissimilar to the nodal-line structures of eigenmodes.

## 3. Maximum Entropy States of the Vibrating Plate

The transverse vibration of a plate is governed by the biharmonic equation [21]

(1) $$\left(\nabla_{2D}^{4}-k^{4}\right)\psi (r)=0,$$

where

(2) $$\nabla_{2D}^{2}=\frac{\partial^{2}}{\partial x^{2}}+\frac{\partial^{2}}{\partial y^{2}},$$

r=(x,y), and *k* is the wave number. Equation (1) can be factorized as

(3) $$\left(\nabla_{2D}^{2}+k^{2}\right)\left(\nabla_{2D}^{2}-k^{2}\right)\psi (r)=0,$$

in which the first factor describes propagating dispersive waves, whereas the second one describes evanescent waves. Since the aspect ratio *h*/*L* is generally less than 0.02, the vibrating eigenmodes can be approximated with the 2D Helmholtz equation [22], where *h* and *L* are the thickness and lateral size of the studied plate, respectively. Even so, the free edge boundary conditions make the problem particularly difficult, as noted by Rayleigh [23]. As a first approximation, one can model the plate as a tightly stretched thin elastic membrane by assuming the constant *m* (the ratio of lateral contraction to longitudinal elongation) to be zero, i.e., every point of the circumference is free to move along lines perpendicular to the plane of the plate. Under this approximation, the boundary condition can be simplified as the Neumann boundary conditions with ∂ψ/∂n=0 on the periphery. Strictly, the Neumann boundary condition is practically applicable to a stretched membrane, but not to a plate vibrating in virtue of rigidity. Nevertheless, it has been demonstrated [24] that a hypothetical free membrane can be used to deduce some of the classical Chladni figures successfully. Based on this confirmation, a free membrane with a point scatter is proposed to model the modern Chladni figures.

The two-dimensional Helmholtz equation for the domain Ω with boundary shape dΩ is given by

(4) $$\left(\nabla_{2D}^{2}+k_{n}^{2}\right)\psi_{n}(r)=0,$$

where $k_{n}$ and $\psi_{n}$ with the indices of n=0,1,2,3⋯ are the eigenvalues and eigenfunctions, respectively. Considering the exciter as a point source, the inhomogeneous Helmholtz equation for characterizing the modern Chladni plate is given by

(5) $$\left(\nabla_{2D}^{2}+k^{2}\right)\Psi (r,r_{s};k)=A\;\delta (r-r_{s}),$$

where $r_{s}$ is the position of the exciter and *A* is the amplitude of the driving force. Using the expansion of the eigenfunctions $\left\{\psi_{n}(r)\right\}$ (n=1,2,3⋯), the source function $\delta (r-r_{s})$ and the response function $\Psi (r,r_{s};k)$ in Equation (5) can be expressed as

(6) $$\delta (r-r_{s})=\underset{N\to \infty }{\lim}\;\sum_{n=0}^{N}\;\psi_{n}^{*}(r_{s})\;\psi_{n}(r),$$

(7) $$\Psi (r,r_{s};k)=\underset{N\to \infty }{\lim}\;\sum_{n=0}^{N}\;a_{n}(r_{s};k)\;\psi_{n}(r),$$

Substituting Equations (6) and (7) into Equation (5) and using Equation (4), the coefficients $a_{n}(r_{s};k)$ in the response function $\Psi (r,r_{s};k)$ can be derived as

(8) $$a_{n}(r_{s};k)=A\frac{\psi_{n}^{*}(r_{s})\;}{k^{2}-k_{n}^{2}}.$$

The response function is given by $\Psi (r,r_{s};k)=A\;G(r,r_{s};k)$, where $G(r,r_{s};k)$ is the well-known Green function

(9) $$G(r,r_{s};k)=\underset{N\to \infty }{\lim}\;\sum_{n=0}^{N}\;\frac{\psi_{n}^{*}(r_{s})\;\psi_{n}(r)}{k^{2}-k_{n}^{2}}.$$

Practically, a finite upper index *N* is chosen for numerical analysis. The use of a finite index *N* physically corresponds to a truncated basis. The truncation of a basis is relevant to a realistic system with a finite range of frequency response. The influence of the value of the index *N* is discussed in all the following numerical analyses.

The resonant frequencies in modern Chladni experiment have been found to be intriguingly correlated to the maximum entropy states [16,19]. From the Shannon theory and in terms of the coefficients $a_{n}(r_{s};k)$, the information entropy of the response function $\Psi (r,r_{s};k)$ can be evaluated by [25,26]

(10) $$S(r_{s};k)=-\sum_{n=0}^{N}p_{n}(r_{s};k)\ln\left[p_{n}(r_{s};k)\right],$$

where the probability of the eigenmode $\psi_{n}(r)$ is given by

(11) $$p_{n}(r_{s};k)=\frac{{\left|a_{n}(r_{s};k)\right|}^{2}}{\sum_{n=0}^{N}{\left|a_{n}(r_{s};k)\right|}^{2}}.$$

For bipartite states, the information entropy is often used to evaluate the degree of spatial entanglement [27,28,29,30]. Here we demonstrate complete results for the correlation between the resonant frequencies and the maximum entropy states for the square and equilateral triangle plates. For a square-shape plate with the region in 0≤x,y≤L, the eigenfunctions are given by

(12) $$\psi_{n,m}(r)=\frac{2}{L}\cos\left(\frac{n\pi }{L}x\right)\cos\left(\frac{m\pi }{L}y\right).$$

The eigenvalue corresponding to the eigenfunction $\psi_{n,m}(r)$ is given by

(13) $$k_{n,m}=\frac{\pi }{L}\sqrt{n^{2}+m^{2}}.$$

Using Equations (8), (10) and (11), the probability of the eigenfunction $\psi_{n,m}(r)$ in the information entropy for a given upper index *N* is given by

(14) $$p_{n,m}(r_{s};k)={\left[\sum_{n=0}^{N}\sum_{m=0}^{N}\;\frac{{\left|\psi_{n,m}(r_{s})\right|}^{2}}{{\left(k^{2}-k_{n,m}^{2}\right)}^{2}}\right]}^{-1}\;\frac{{\left|\psi_{n,m}(r_{s})\right|}^{2}}{{\left(k^{2}-k_{n,m}^{2}\right)}^{2}}.$$

In terms of the probabilities $p_{n,m}(r_{s};k)$, the information entropy can be expressed as

(15) $$S(r_{s};k)=-\sum_{n=0}^{N}\sum_{m=0}^{N}p_{n,m}(r_{s};k)\ln\left[p_{n,m}(r_{s};k)\right].$$

For an equilateral triangle plate with vertices at (0, 0), $\left(L/2,\;\sqrt{3}L/2\right)$, and $\left(-L/2,\;\sqrt{3}L/2\right)$, the eigenmodes for the driving source with even symmetry can be expressed as [31]

(16) $$\begin{array}{ll} {\tilde{\psi }}_{n,m}(r)=\sqrt{\frac{16}{L^{2}3\sqrt{3}}}\{\cos & \left[\frac{2\pi }{3L}(2n-m)x\right]\cos\left(\frac{2\pi }{\sqrt{3}L}m\;y\right)\\ & +\cos\left[\frac{2\pi }{3L}(2m-n)x\right]\cos\left(\frac{2\pi }{\sqrt{3}L}n\;y\right)\\ & +\cos\left[\frac{2\pi }{3L}(n+m)x\right]\cos\left[\frac{2\pi }{\sqrt{3}L}(n-m)y\right]\} \end{array}$$

for m≥2n. The eigenvalue for the eigenmode ${\tilde{\psi }}_{n,m}(r)$ is given by

(17) $${\tilde{k}}_{n,m}=\frac{4\pi }{3L}\sqrt{n^{2}+m^{2}-n\;m}.$$

The information entropy for the equilateral triangle plate with an upper index *N* is given by

(18) $$S(r_{s};k)=-\sum_{n=0}^{N}\sum_{m=2n}^{N}p_{n,m}(r_{s};k)\ln\left[p_{n,m}(r_{s};k)\right],$$

(19) $$p_{n,m}(r_{s};k)={\left[\sum_{n=0}^{N}\sum_{m=2n}^{N}\frac{{\left|{\tilde{\psi }}_{n,m}(r_{s})\right|}^{2}}{{\left(k^{2}-{\tilde{k}}_{n,m}^{2}\right)}^{2}}\right]}^{-1}\;\frac{{\left|{\tilde{\psi }}_{n,m}(r_{s})\right|}^{2}}{{\left(k^{2}-{\tilde{k}}_{n,m}^{2}\right)}^{2}}.$$

The information entropy can be exploited to evaluate the effective number $N_{eff}$ of participated eigenfunctions in the response function, given by $N_{eff}=\exp[S(r_{s};k)]$. It has been verified that the resonant wave numbers can be properly attained from local maxima of the spectrum *N_eff_* [16,19].

**Figure 4:**
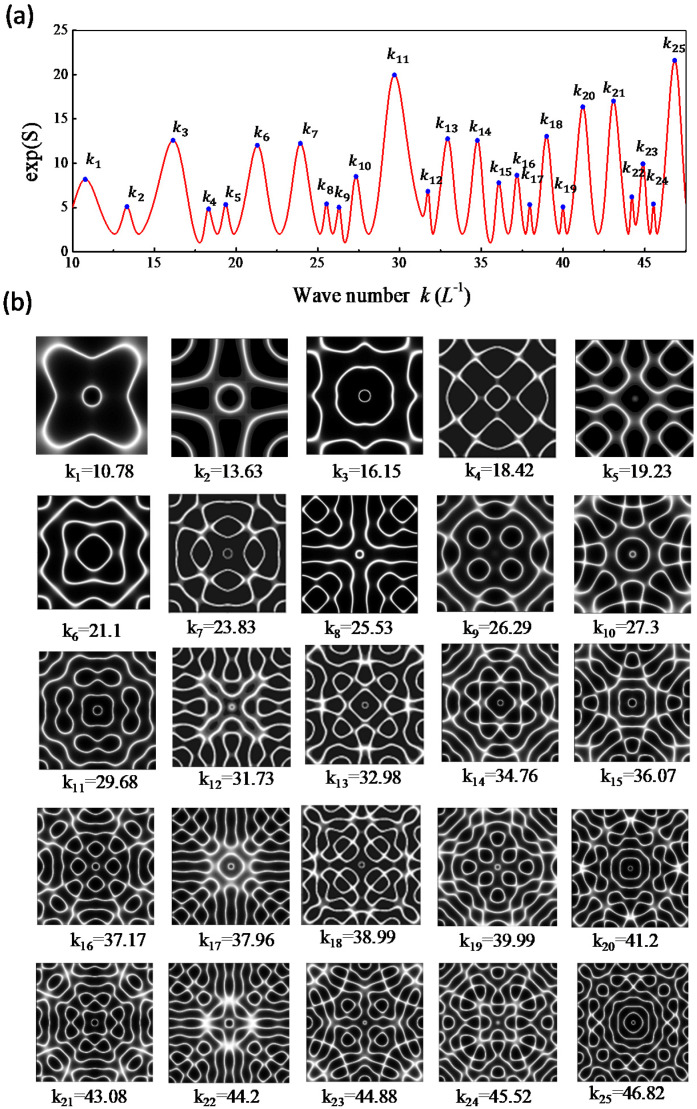
(**a**) Calculated results for $\exp[S(r_{s};k)]$ as a function of *k* for the square plate with $r_{s}=(L/2,L/2)$. (**b**) Nodal-line patterns corresponding to entropy states with local maxima.

**Figure 5:**
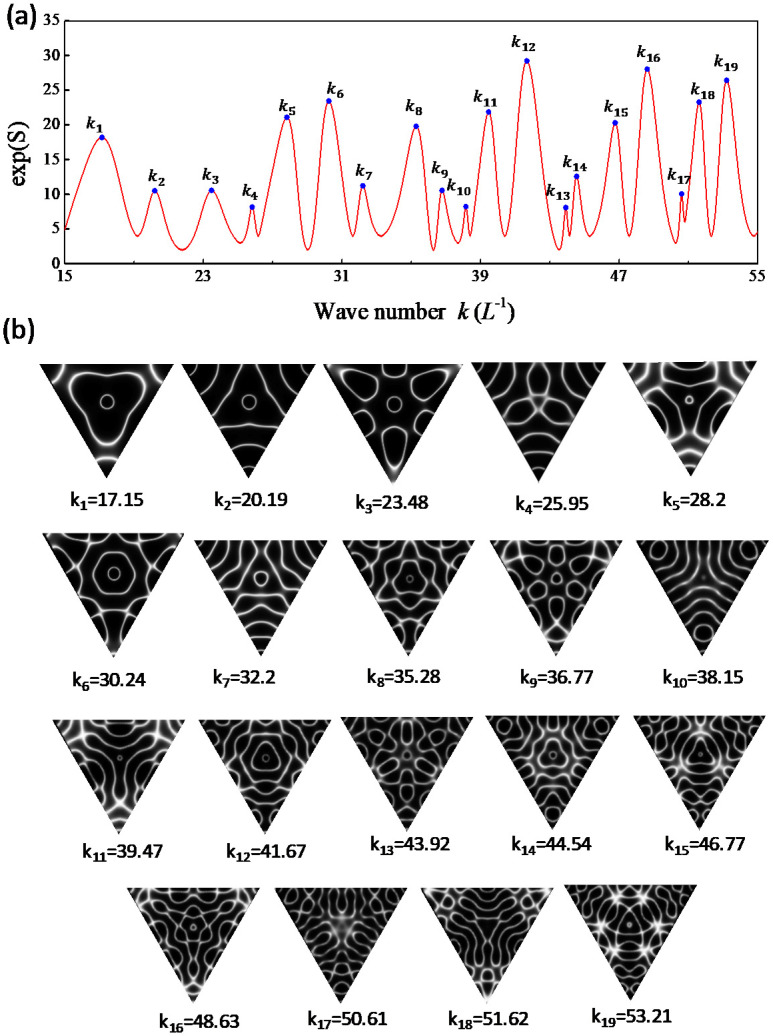
(**a**) Calculated results for $\exp[S(r_{s};k)]$ as a function of *k* for the equilateral triangle plate with $r_{s}=(0,L/\sqrt{3})$. (**b**) Nodal-line patterns corresponding to entropy states with local maxima.

Based on thorough computation, the entropy $S(r_{s};k)$ is confirmed to be nearly independent of the choice of the upper index *N* on condition that N>kL is satisfied. Since the range of *kL* for investigation is less than 60, the results obtained with N=100 are used for convenience. Figure 4a depicts the calculated results for $\exp[S(r_{s};k)]$ as a function of *k* with $r_{s}=(L/2,L/2)$. The nodal-line patterns corresponding to the local maxima are shown completely in Figure 4b. The nodal-line patterns can be one-to-one seen to agreeably resemble the experimental results for the resonant modes shown in Figure 2b. Not only the square plate, the same correspondence between the resonant frequencies and the maximum entropy states can be also confirmed for the equilateral triangle plate, as shown in Figure 5a,b for calculated results with $r_{s}=(0,L/\sqrt{3})$.

Moreover, the dispersion relation for the flexural wave of the plate can be directly deduced from the correspondence between the resonant frequencies *f* and the resonant wave numbers *k* of maximum entropy states. From the Kirchhoff–Love plate theory [32], the dispersion relation between *f* and *k* is given by $f(k)=Ck^{2}/2\pi$, where $C=\sqrt{D/\rho h}$, *D* is the flexural rigidity given by $D=Eh^{3}/[12(1-\nu^{2})]$, *E* is the Young’s modulus, *ν* is the Poisson ratio, *ρ* is the mass density, and *h* is the thickness of plate. By using the material properties of *E =* 70 GPa, *ν =* 0.33, *ρ =* 2700 kg/m^3^ [33], and *h* = 1 mm, the theoretical coefficient C/2π can be found to be 0.248. From the correspondence between the resonant frequencies and the maximum entropy states, the coefficient C/2π can be found to be approximately 0.226 and 0.220 for the square and equilateral triangle plates, respectively. The good agreement confirms that the information of the flexural rigidity *D* and the Young’s modulus *E* can be deduced by using the maximum entropy principle as well as the dispersion relation.

The maximum entropy principle has been developed to analyze the collective behavior in multimode systems such as maximum emission for lasers [34], self-organization for complex systems [35], wave function localization for disordered systems [36], and phase transitions for open quantum systems [37]. However, the coincidence of the resonant frequencies and the maximum entropy states can be comprehended from the principle of energy equipartition in statistical mechanics. Nevertheless, it is of great importance to disclose the origin of the resonant modes in modern Chladni plates for developing further applications.

## 4. Coupling Interaction between Source and Plate

Considering the mechanical oscillator to play as not only a driving source but also a coupling interaction, the characteristic equation in Equation (5) is modified as

(20) $$\left(\nabla_{2D}^{2}+k^{2}+\alpha \delta (r-r_{s})\right)\Psi_{\alpha }(r,r_{s};k)=A\;\delta (r-r_{s}),$$

where the parameter *α* is a real number. The term $\alpha \;\delta (r-r_{s})\Psi_{\alpha }(r,r_{s};k)$ in Equation (20) is to take account of the coupling effect between the mechanical oscillator and the thin plate. The coupling interaction comes from the reaction force of the thin plate subjected to the driving force. The dimensionless parameter *α* is directly related to the coupling strength. Intuitionally, the coupling strength is proportional to the relative tightness of the fixed force between the plate and the oscillator. The point interaction [38,39,40] has attracted much attention in the research of the nuclear [41], atomic [42], solid-state [43], and particle physics [44]. Furthermore, Šeba [45] studied the coupling interaction of a delta-function potential in two-dimensional integrable billiards to verify that the strong coupling can cause the transition from integrable to chaotic feature [46,47,48,49,50,51,52,53]. On the other hand, a novel method for analytically solving field distribution in two-dimensional inhomogeneous waveguides is presented in Ref. [54].

Using the expansion forms in Equations (6) and (7), the response function $\Psi_{\alpha }(r,r_{s};k)$ in Equation (20) can be derived as

(21) $$\Psi_{\alpha }(r,r_{s};k)=\frac{A\;G(r,r_{s};k)}{1+\alpha G(r_{s},r_{s};k)}$$

where $G(r_{s},r_{s};k)$ is a meromorphic function. Note that the identification of $\delta (r-r_{s})\Psi_{\alpha }(r,r_{s};k)=\delta (r-r_{s})\Psi_{\alpha }(r_{s},r_{s};k)$ has been used in the derivation of Equation (21). The norm $\left|\Psi_{\alpha }(r_{s},r_{s};k)\right|$ indicates the response amplitude of the plate at the driving position $r_{s}$ for a given wave number *k*. Consequently, the local maxima of $\left|\Psi_{\alpha }(r_{s},r_{s};k)\right|$ as a function of the driving wave number *k* can be identified to correspond to the resonant wave numbers for the modern Chladni plate locally excited at the position $r_{s}$. From Equation (21), the amplitude of the response function $\left|\Psi_{\alpha }(r_{s},r_{s};k)\right|$ can be expressed as

(22) $$\left|\Psi_{\alpha }(r_{s},r_{s};k)\right|=\left|\frac{A\;G(r_{s},r_{s};k)}{1+\alpha \;G(r_{s},r_{s};k)}\right|.$$

Equation (22) indicates that if $G(r_{s},r_{s};k)\neq 0$, the resonant wave numbers corresponding to the local maxima of $\left|\Psi_{\alpha }(r_{s},r_{s};k)\right|$ are determined by the transcendental equation $1+\alpha \;G(r_{s},r_{s};k)=0$. Note that the overall structure of the distribution $\left|\Psi_{\alpha }(r_{s},r_{s};k)\right|$ is independent of the driving amplitude *A*. Without the coupling effect for the case α=0, the resonant wave numbers are determined by the poles of $G(r_{s},r_{s};k)$, which are exactly the eigenvalues $k_{n}$ of the free plates. For a given coupling factor α, the resonant wave numbers are determined from $G(r_{s},r_{s};k)=-1/\alpha$. The key issue to validate the present model is to verify that the distribution $\left|\Psi_{\alpha }(r_{s},r_{s};k)\right|$ can be consistent with the experimental spectrum as well as the maximum entropy states.

**Figure 6:**
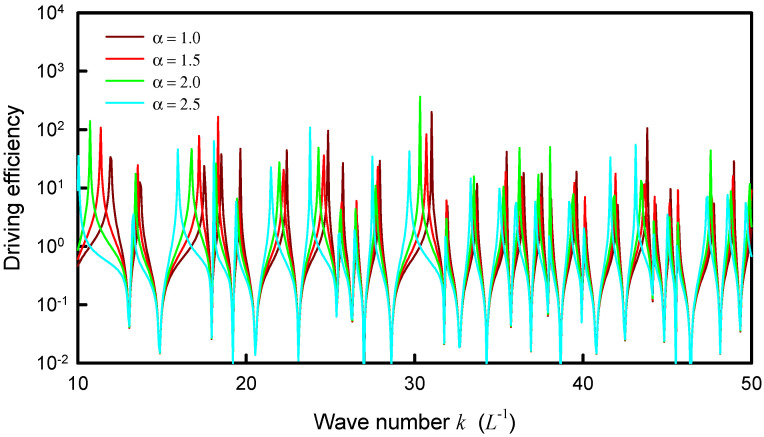
Calculated result for $\left|\Psi_{\alpha }(r_{s},r_{s};k)\right|$ by using Equation (22) as a function of *k* for the square plate with $r_{s}=(L/2,L/2)$ and *N* = 50 for several different coupling factors α = 1.0, 1.5, 2.0 and 2.5.

**Figure 7:**
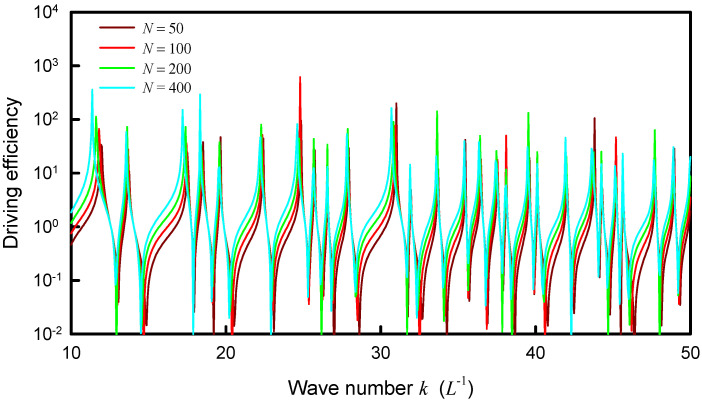
Dependence of the distribution $\left|\Psi_{\alpha }(r_{s},r_{s};k)\right|$ on the upper index *N* for the square plate with a fixed α=1.0.

Unlike the numerical analyses for information entropy, the calculated results for the distribution $\left|\Psi_{\alpha }(r_{s},r_{s};k)\right|$ are found to be not only dependent on the coupling factor α but also on the upper index *N*. Nevertheless, the asymptotic behavior for the index *N* can be attained from the computation. The dependence of the distribution $\left|\Psi_{\alpha }(r_{s},r_{s};k)\right|$ on the coupling factor α is first discussed. Figure 6 shows the calculated result for $\left|\Psi_{\alpha }(r_{s},r_{s};k)\right|$ by using Equation (22) as a function of *k* for the square plate with $r_{s}=(L/2,L/2)$ and *N* = 50 for several different coupling factors α = 1.0, 1.5, 2.0 and 2.5. The coupling factor α can be clearly seen to cause the redshifts of the resonant wave numbers. The larger the coupling factor, the greater the redshift. On the other hand, the dependence of the distribution $\left|\Psi_{\alpha }(r_{s},r_{s};k)\right|$ on the upper index *N* is shown in Figure 7 for the square plate with a fixed α=1.0. The overall distribution $\left|\Psi_{\alpha }(r_{s},r_{s};k)\right|$ can be found to be a little redshifted with increasing the upper index *N*.

**Figure 8:**
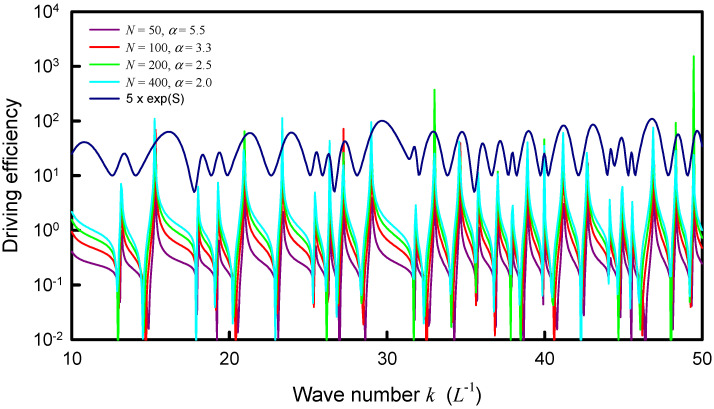
Calculated distribution $\left|\Psi_{\alpha }(r_{s},r_{s};k)\right|$ for the square plate with nearly the same resonant peaks by using different upper indices *N* companioned with different coupling factors α.

**Figure 9:**
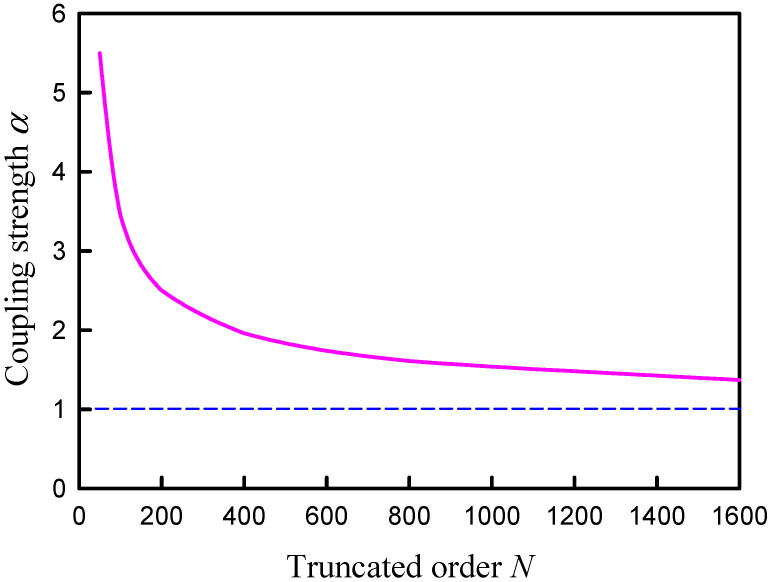
Numerical result for the relationship between the upper index *N* and the effective coupling factor α for the distributions $\left|\Psi_{\alpha }(r_{s},r_{s};k)\right|$ with the best fit to the maximum entropy states.

**Figure 10:**
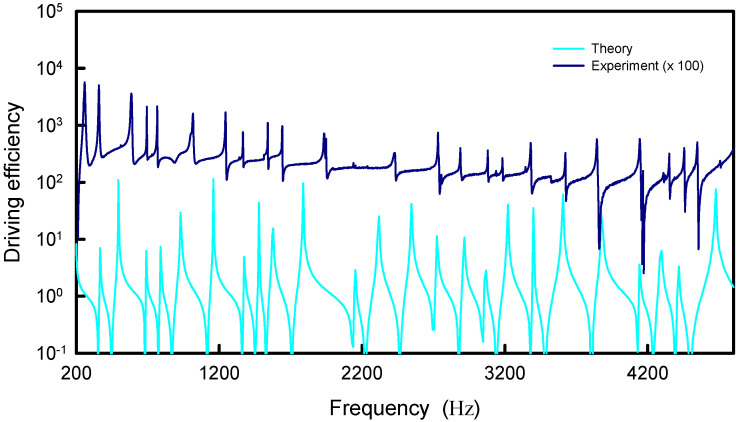
Calculated result for the distribution $\left|\Psi_{\alpha }(r_{s},r_{s};k)\right|$ with N=400 and α=2.0 for the square plate to compare with the experimental spectrum.

The resonant wave numbers are confirmed to approach some asymptote for increasing the upper index *N*. Numerical analyses reveal that as N→∞, the coupling factor of α=1.0 can make the distribution $\left|\Psi_{\alpha }(r_{s},r_{s};k)\right|$ agree the best with the experimental spectrum as well as the maximum entropy states. In practice, when a smaller upper index *N* is used in the calculation, a larger value for the coupling factor α can be effectively utilized to lead the distribution $\left|\Psi_{\alpha }(r_{s},r_{s};k)\right|$ to be nearly the same, as shown in Figure 8. The resonant peaks of the distributions $\left|\Psi_{\alpha }(r_{s},r_{s};k)\right|$ for different upper index *N* can be seen to excellently coincide together. More importantly, all resonant peaks agree very well with the maximum entropy states. In Figure 8, the information entropy is multiplied by a factor of 5 for convenience of displaying the comparison. Figure 9 shows the numerical result for the relationship between the upper index *N* and the effective coupling factor α for the distributions $\left|\Psi_{\alpha }(r_{s},r_{s};k)\right|$ with the best fit to the maximum entropy states. By using the deduced dispersion relation in Section 3, the distribution $\left|\Psi_{\alpha }(r_{s},r_{s};k)\right|$ with N=400 and α=2.0 is plotted in Figure 10 to compare with the experimental spectrum. For clear comparison, the experimental spectrum is multiplied by a factor of 100. The theoretical resonant spectrum generally agrees very well with the experimental result. A little discrepancy is mainly attributed to the simplified model for the dispersion relation between *f* and *k*.

**Figure 11:**
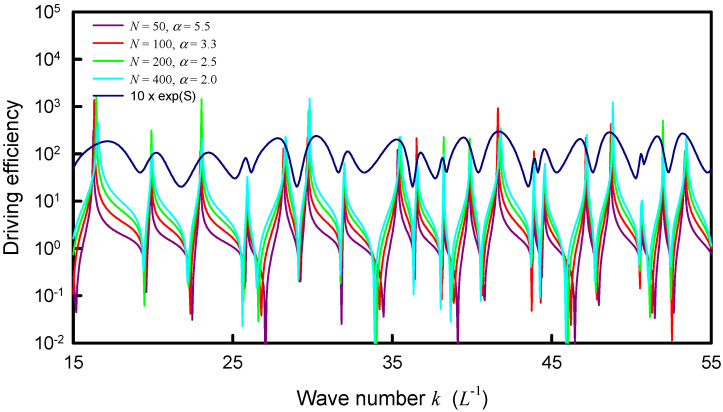
Calculated distribution $\left|\Psi_{\alpha }(r_{s},r_{s};k)\right|$ for the equilateral triangle plate with nearly the same resonant peaks by using different upper indices *N* companioned with different coupling factors α.

**Figure 12:**
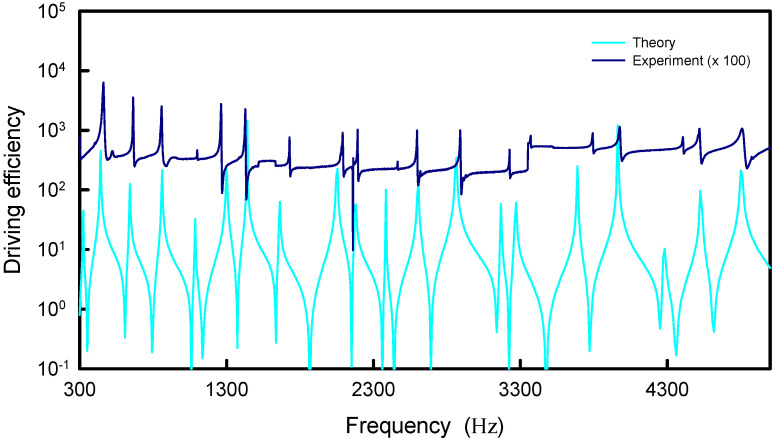
Calculated result for the distribution $\left|\Psi_{\alpha }(r_{s},r_{s};k)\right|$ with N=400 and α=2.0 for the equilateral triangle plate to compare with the experimental spectrum.

With the best fit to the maximum entropy states for the equilateral triangle plate, the relationship between the upper index *N* and the effective coupling factor α for the distributions $\left|\Psi_{\alpha }(r_{s},r_{s};k)\right|$ is intriguingly found to be almost the same as the case of square plate. Figure 11 depicts the calculated results for the distributions $\left|\Psi_{\alpha }(r_{s},r_{s};k)\right|$ with four different best pairs of *N* and α to compare with the distribution of the information entropy. For convenience of displaying the comparison, the information entropy is multiplied by a factor of 10. Just like the plot in Figure 8, the peaks of the distributions $\left|\Psi_{\alpha }(r_{s},r_{s};k)\right|$ can be clearly seen to coincide together and to agree quite well with the maximum entropy states. By using the deduced dispersion relation in Section 3, the comparison between the experimental spectrum and the distribution $\left|\Psi_{\alpha }(r_{s},r_{s};k)\right|$ with N=400 and α=2.0 is shown in Figure 12. For clear comparison, the experimental spectrum is multiplied by a factor of 100. Once again, the theoretical resonant spectrum is in good agreement with the experimental result. The good agreement further confirms that the point-interaction model can be used to manifest the formation of modern Chladni patterns.

## 5. Conclusions

In summary, we have demonstrated that the resonant modes in the modern Chladni experiment can be completely reconstructed with the maximum entropy states solved from the inhomogeneous Helmholtz equation for the square and equilateral triangle plates. We have judiciously considered the point interaction coming from the driving oscillator to explore the relevance between the maximum entropy states and experimental resonant modes. A dimensionless factor α and a truncated basis with an upper index *N* were used to characterize the coupling strength and the δ potential for the point interaction. We have employed an asymptotic way to verify that the coupling strength of α=1.0 can make the theoretical resonant modes agree very well with the maximum entropy states as N→∞. We have further validated that a larger coupling strength α can lead to nearly the same resonant spectrum when a smaller upper index *N* is used in the calculation.

## Data Availability

All of the data reported in the paper are presented in the main text. Any other data will be provided on request.

## References

[1] Chladni E.F.F. (1787). Entdeckungen über die Theorie des Klanges.

[2] Chladni E.F.F. (1802). Die Akustik.

[3] Jain S.R., Samajdar R. (2017). Nodal portraits of quantum billiards: Domains, lines, and statistics. Rev. Mod. Phys..

[4] Dorrestijn M., Bietsch A., Açıkalın T., Raman A., Hegner M., Meyer E., Gerber C. (2007). Chladni figures revisited based on nanomechanics. Phys. Rev. Lett..

[5] Taillan C., Combe N., Morillo J. (2011). Nanoscale self-organization using standing surface acoustic waves. Phys. Rev. Lett..

[6] Friend J., Yeo L.Y. (2011). Microscale acoustofluidics: Microfluidics driven via acoustics and ultrasonics. Rev. Mod. Phys..

[7] Mak S.Y., Li Z., Frere A., Chan T.C., Shum H.C. (2014). Musical interfaces: Visualization and reconstruction of music with a microfluidic two-phase flow. Sci. Rep..

[8] Ohlin K., Berggren K.F. (2016). Patterns beyond Faraday waves: Observation of parametric crossover from Faraday instabilities to the formation of vortex lattices in open dual fluid strata. Eur. J. Phys..

[9] Misseroni D., Colquitt D.J., Movchan A.B., Movchan N.V., Jones I.S. (2016). Cymatics for the cloaking of flexural vibrations in a structured plate. Sci. Rep..

[10] Scholz C., Engel M., Pöschel T. (2018). Rotating robots move collectively and self-organize. Nat. Commun..

[11] Scholz C., Pöschel T. (2017). Velocity distribution of a homogeneously driven two-dimensional granular gas. Phys. Rev. Lett..

[12] Collins D.J., Morahan B., Garcia-Bustos J., Doerig C., Plebanski M., Neild A. (2015). Two-dimensional single-cell patterning with once cell per well driven by surface acoustic waves. Nat. Commun..

[13] Ding X., Lin S., Kiraly B., Yue H., Li S., Chiang I.K., Shi J., Benkovic S., Huang T. (2012). On-chip manipulation of single microparticles, cells, and organisms using surface acoustic waves. Proc. Natl Acad. Sci. USA.

[14] Zhou Q., Sariola V., Latifi K., Liimatainen V. (2016). Controlling the motion of multiple objects on a Chladni plate. Nat. Commun..

[15] Whitehill J., Neild A., Ng T.W., Strokes M. (2010). Collection of suspended particles in a drop using low frequency vibration. Appl. Phys. Lett..

[16] Tuan P.H., Wen C.P., Chiang P.Y., Yu Y.T., Liang H.C., Huang K.F., Chen Y.F. (2015). Exploring the resonant vibration of thin plates: Reconstruction of Chladni patterns and determination of resonant wave numbers. J. Acoust. Soc. Am..

[17] Tuan P.H., Liang H.C., Tung J.C., Chiang P.Y., Huang K.F., Chen Y.F. (2015). Manifesting the evolution of eigenstates from quantum billiards to singular billiards in the strongly coupled limit with a truncated basis by using RLC networks. Phys. Rev. E.

[18] Tuan P.H., Tung J.C., Liang H.C., Chiang P.Y., Huang K.F., Chen Y.F. (2015). Resolving the formation of modern Chladni figures. Europhys. Lett..

[19] Tuan P.H., Lai Y.H., Wen C.P., Huang K.F., Chen Y.F. (2018). Point-driven modern Chladni figures with symmetry breaking. Sci. Rep..

[20] Tuan P.H., Wen C.P., Yu Y.T., Liang H.C., Huang K.F., Chen Y.F. (2014). Exploring the distinction between experimental resonant modes and theoretical eigenmodes: From vibrating plates to laser cavities. Phys. Rev. E.

[21] Chakraverty S. (2009). Vibration of Plates.

[22] Ventsel E., Krauthammer T. (2004). Thin Plates and Shells.

[23] Rayleigh J.W.S. (1945). Theory of Sound.

[24] Waller M.D. (1939). Vibrations of free square plates: Part I. Normal vibrating modes. Proc. Phys. Soc..

[25] Shannon C.E. (1951). Prediction and entropy of printed English. Bell Syst. Tech. J..

[26] Jaynes E.T. (1957). Information theory and statistical mechanics. Phys. Rev..

[27] Ekert A., Knight P.L. (1995). Entangled quantum systems and the Schmidt decomposition. Am. J. Phys..

[28] Law C.K., Eberly J.H. (2004). Analysis and interpretation of high transverse entanglement in optical parametric down conversion. Phys. Rev. Lett..

[29] Law C.K., Walmsley I.A., Eberly J.H. (2000). Continuous frequency entanglement: Effective finite Hilbert space and entropy control. Phys. Rev. Lett..

[30] Fedorov M.V., Miklin N.I. (2014). Schmidt modes and entanglement. Contemp. Phys..

[31] Práger M. (1998). Eigenvalues and eigenfunctions of the Laplace operator on an equilateral triangle. Appl. Math..

[32] Leissa A.W. (1993). Vibration of Plates.

[33] Van Vlack L.H. (1980). Elements of Material Science and Engineering.

[34] Tang C.L., Statz H. (1967). Maximum-emission principle and phase locking in multimode lasers. J. Appl. Phys..

[35] Haken H. (2006). Information and Self-Organization: A Macroscopic Approach to Complex Systems.

[36] Heller E.J. (1987). Quantum localization and the rate of exploration of phase space. Phys. Rev. A.

[37] Jung C., Müller M., Rotter I. (1999). Phase transitions in open quantum systems. Phys. Rev. E.

[38] Albeverio S., Gesztesy F., Høegh-Krohn R., Holden H. (2012). Solvable Models in Quantum Mechanics.

[39] Schmidt A.G.M., Cheng B.K., da Luz M.G.E. (2002). Green functions for generalized point interactions in one dimension: A scattering approach. Phys. Rev. A.

[40] Arnbak H., Christiansen P.L., Gaididei Y.B. (2011). Non-relativistic and relativistic scattering by short-range potentials. Philos. Trans. R. Soc. A.

[41] Kruppa A.T., Varga K., Révai J. (2001). Local realizations of contact interactions in two-and three-body problems. Phys. Rev. C.

[42] Demkov Y., Ostrovskii V.N. (1989). Zero-Range Potentials and Their Applications in Atomic Physics.

[43] Doniach S., Sondheimer E. (1998). Green’s Functions for Solid State Physicists.

[44] Thorn C. (1979). Quark confinement in the infinite-momentum frame. Phys. Rev. D.

[45] Šeba P. (1990). Wave chaos in singular quantum billiard. Phys. Rev. Lett..

[46] Shigehara T. (1994). Conditions for the appearance of wave chaos in quantum singular systems with a pointlike scatterer. Phys. Rev. E.

[47] Šeba P., Exner P. (1996). Point interactions in two and three dimensions as models of small scatterers. Phys. Lett. A.

[48] Bogomolny E., Gerland U., Schmit C. (2001). Singular statistics. Phys. Rev. E.

[49] Berkolaiko G., Keating J.P., Winn B. (2003). Intermediate wave function statistics. Phys. Rev. Lett..

[50] Tudorovskiy T., Kuhl U., Stöckmann H.J. (2010). Singular statistics revised. New J. Phys..

[51] Rudnick Z., Ueberschär H. (2012). Statistics of wave functions for a point scatterer on the torus. Commun. Math. Phys..

[52] Weaver R.L., Sornette D. (1995). Range of spectral correlations in pseudointegrable systems: Gaussian-orthogonal-ensemble statistics in a rectangular membrane with a point scatterer. Phys. Rev. E.

[53] Shigehara T., Cheon T. (1996). Wave chaos in quantum billiards with a small but finite-size scatterer. Phys. Rev. E.

[54] Grica T., Eldlioa M., Cada M., Pistor J. (2015). Analytic solution to field distribution in two-dimensional inhomogeneous waveguides. J. Electromagn. Waves Appl..

